# Cross-sectional atlas of the tree shrew (*Tupaia belangeri*) head, neck, and trunk: a comparison of micro-CT imaging and cadaveric sections

**DOI:** 10.3389/fvets.2026.1755634

**Published:** 2026-02-25

**Authors:** Fangfang Chen, Yiwei Feng, Nan Shi, Pengcheng Zhao, Zongjian Huang, Yu Ren, Wei Xia, Anzhou Tang

**Affiliations:** 1 Department of Otorhinolaryngology Head and Neck Surgery, The First Affiliated Hospital of Guangxi Medical University, Nanning, Guangxi, China; 2 Key Laboratory of Early Prevention and Treatment for Regional High-Frequency Tumours (Guangxi Medical University), Ministry of Education, Nanning, China; 3 Department of Otorhinolaryngology, Head and Neck Surgery, Zhongnan Hospital of Wuhan University, Wuhan, China

**Keywords:** cadaveric sections, cross-sectional anatomy, head-neck, micro-computed tomography, tree shrew

## Abstract

**Introduction:**

Tree shrews are widely used as animal models in various research fields, however, a comprehensive cross-sectional anatomic reference for this species is currently lacking.

**Methods:**

In this study, Conventional Micro-CT scanning (40 μm slice thickness) was performed on two healthy adult tree shrews to obtain head-neck-thorax-abdomen cross-sectional images. Upon completion of the CT session, the tree shrew was euthanized and transferred to a −20 °C freezer until completely frozen maintaining the same position the plastic support. Subsequently, the specimen was transversely sectioned from the tip of the nose to the anus at 5-mm intervals using an electric bone saw. The CT images were aligned with the frozen anatomical sections, and significant structures were identified and labeled with reference to the relevant literature.

**Results:**

This study provides an atlas of normal cross-sectional gross and CT anatomy of the tree shrew head, neck, thorax and abdomen, consisting of 18 anatomical slices and the corresponding CT images and the nomenclature list for each image.

**Conclusions:**

This study provides an anatomic and Micro-CT atlas of the head, neck, thorax and abdomen of the tree shrew, which researchers can use as a reference for the interpretation of any cross-sectional imaging modality in tree shrews.

## Introduction

Tree shrew (*Tupaia belangeri* Chinensis) is a small mammal resembling a squirrel and is widely distributed in Southeast Asia. The adult body weight ranges from 120 to 150 g, the body length is 12 to 24 cm, and the tail is approximately equal in length to the body. The tree shrew has been proposed as a laboratory animal because it is small, reproduces quickly, and is easy to capture. Recent studies have confirmed that tree shrews share a high degree of similarity with humans in anatomy, genetic characteristics, and metabolism. Taxonomically, tree shrews belong to the order Scandentia, which is more closely related to primates (and thus to humans) than common laboratory animals (1–4). Therefore, tree shrews are widely used in the field of biomedical research (5–10). Although the use of tree shrew in experimental research has increased in recent years, anatomical studies on this species are rarely reported compared to those on common laboratory animals such as mice, rats, and rabbits.

Currently, no comprehensive anatomical atlas of the tree shrew is available as a reference, which has become a bottleneck limiting the use of tree shrews in biomedical research. This shortfall further restricts the use of tree shrews as animal models for human diseases. Conventional imaging methods such as ultrasound, computed tomography (CT), and magnetic resonance imaging (MRI) are widely used imaging modalities in medical research. They allow researchers to visualize and interpret cross-sectional images *in vivo* without causing any harm to the animals. However, prior to employing any of the above imaging modalities as diagnostic tools, it is necessary to understand the normal cross-sectional anatomy of the species.

Although cross-sectional anatomical atlases exist for mice, rabbits, dogs, and cats (11–14), there is currently a lack of such a reference for tree shrews. The available literature provides only a few anatomical reference data for gross organs and some cross-sectional images of the brain (15–17). Therefore, this study aimed to provide researchers with a series of cross-sectional cadaver photographs of the head, neck, thorax, and abdomen of normal tree shrews, along with corresponding micro-CT images, to serve as an anatomical reference.

## Materials and methods

A total of four tree shrews (all males, weighing between 130 and 140 g) were used for this study. All the tree shrews were obtained from Kunming zoology Institute, Chinese Academy of Science, and were housed in the Experimental Animal Center of Guangxi Medical University for at least 4 weeks. This study was approved by the Ethics Committee of Guangxi Medical University (Approval No. 201911063). All the animal experiments were performed in accordance with Guangxi Medical University guidelines and the “3 R” principles of animal welfare. Two tree shrews (No. 1 and No. 2) were euthanized via intraperitoneal injection of 2% sodium pentobarbital. A gross dissection of these animals was performed to examine the relationships between different anatomical structures.

Another two tree shrews (No. 3 and No. 4) were anesthetized with an intraperitoneal injection of 2% sodium pentobarbital (60 mg/kg). To prevent the saw blade from overheating during slicing, the fur on the head, neck, and trunk were shaved off with an electric clipper, and the animals were covered with wet gauze to keep them cool. The animals were then placed prone on a plastic support with all four limbs splayed. Micro-CT scanning (Aloka LaTheta LCT 200, Tokyo, Japan) was performed at 60 kV with an anode current of 0.5 mA, obtaining serial transverse images with a slice thickness of 40 μm from the tip of the nose to the anus. Scans were completed over 360° of rotation of the x-ray tube.

After completing the micro-CT scans, animals 3 and 4 were euthanized with an intraperitoneal injection of 2% sodium pentobarbital. The cadavers were fixed in the prone position on plastic support and stored in a freezer at −20 °C. After 24 h, they were removed from the support and transversely sectioned from the tip of the nose to the anus at 5 mm intervals using an electric bone saw. The chosen cryo-section thickness was justified based on a balance between anatomical fidelity and tissue sectioning instrument, allowing precise alignment of sections with the corresponding micro-CT datasets while minimizing tissue deformation. The slices were labeled and refrozen at −20 °C for 72 h to allow excess surface moisture to evaporate. The sections were then photographed from the cranial aspect. The sections from tree shrew No. 3 were preserved at −20 °C for future studies. The vertebral column of tree shrew No. 4 was isolated to identify the vertebral level corresponding to each cross-sectional slice.

Some of the photographs were flipped horizontally, to ensure that the animal's right side corresponds to the viewer's right side, with the animal's ventral side oriented at the bottom of the image. Using anatomical reference materials (11, 18), we identified the organs, major bones, blood vessels, and muscles in each cadaver section and labeled them on the cross-sectional images. The identified structures in each cadaver section were subsequently correlated with their counterparts in the corresponding micro-CT images. The image dimensions were adjusted so that the cadaver photograph and its corresponding CT image were at the same scale, making their cross-sectional areas comparable.

## Results

**Figure 1:**
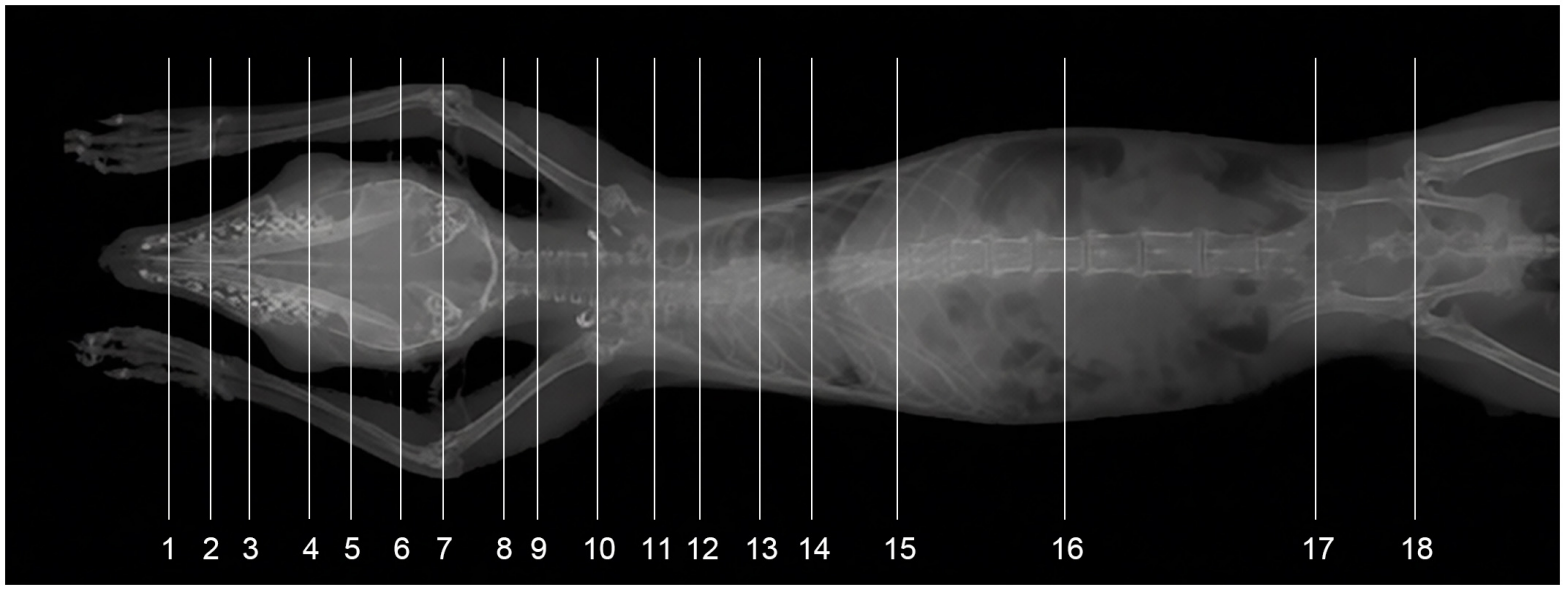
The X-ray photograph of tree shrew total-body. Line 1–18 indicate the approximate levels of each anatomic slice of the frozen cadaver and the contiguous computed tomographic images.

**Figure 2:**
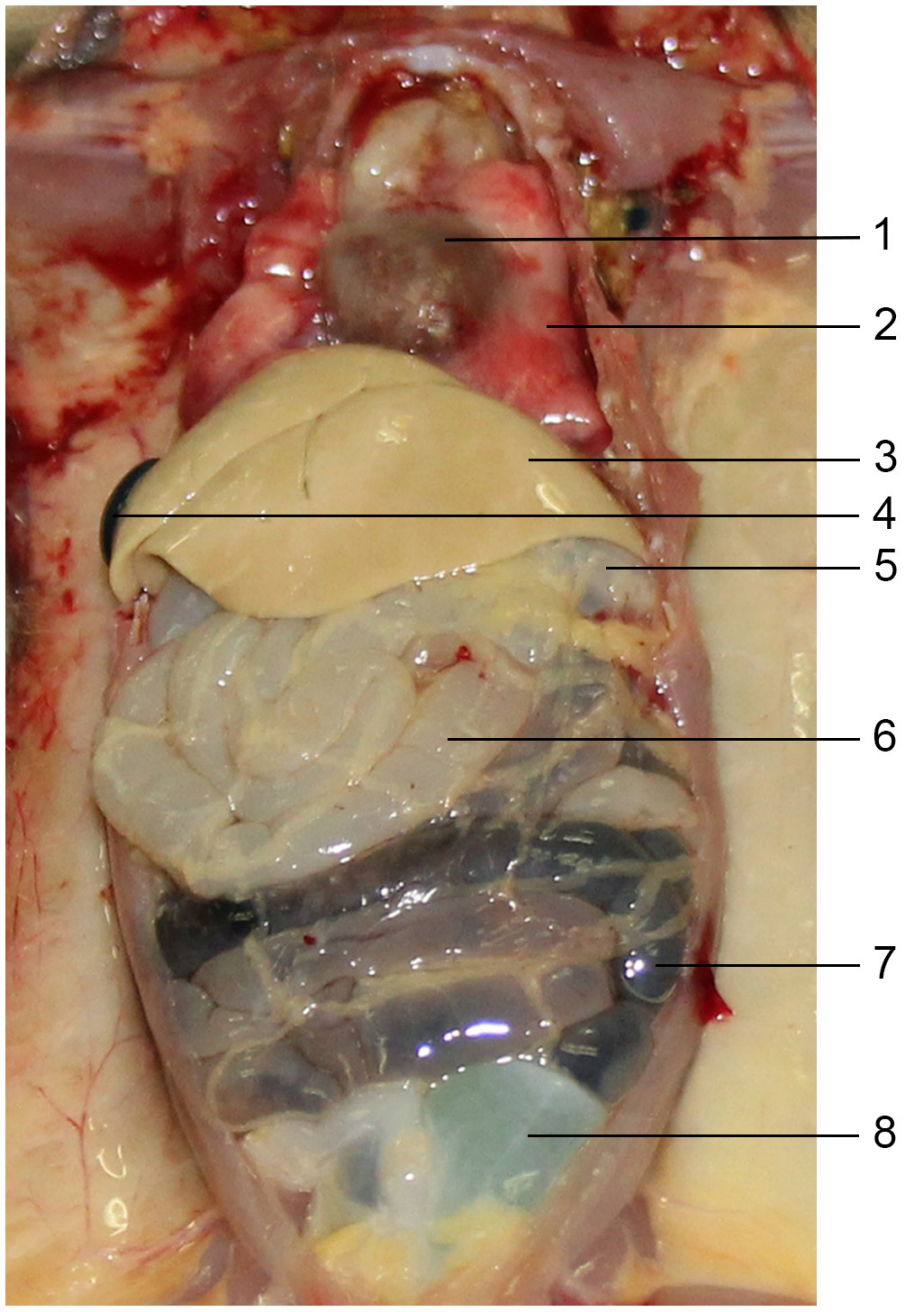
The gross anatomical images of the chest and abdomen of the tree shrew. 1. Heart; 2. Lung; 3. Liver; 4. Gallbladder; 5. Stomach; 6. Small intestine; 7. Colon; 8. Urinary bladder.

The tree shrew specimen was thin and slender, with a cone-shaped skull. The vertebral column consisted of seven cervical, 13 thoracic, six lumbar, three sacral and several caudal. There were 13 pairs of ribs attached to the 13 thoracic vertebrae, of which 10 pairs were true ribs and three pairs were floating ribs (Figure 1). The gross anatomical diagrams of the major organs in the chest and abdomen of the tree shrew are shown in Figure 2. The right lung consisted of four lobes and the left lung had three lobes. The heart, positioned slightly to the left, was enveloped by the lobes of both lungs and by the diaphragm. The liver was located just caudal to the diaphragm and consisted of right, middle, and left lobes. An irregularly shaped caudate lobe protruded from the base of the right lobe. The liver occupied the anterior part of the abdominal cavity and enveloped the stomach in an umbrella-like manner. The gallbladder was large and superficially located; its fundus lay close to the abdominal wall and its body was nestled against the middle lobe of the liver. The right kidney was located below and to the right of the stomach. The small intestine consisted of the duodenum, jejunum, and ileum, which were all connected by the mesentery; however, the boundary between these segments were not distinct. There was a clear boundary between the ileum and cecum. The distal end of the cecum was elongated and free from the abdominal wall and the ileum. A dilated urinary bladder was observed below the cecum, and no appendix was found. The proximal cecum continued into the colon, which could be subdivided into ascending, transverse, and descending segments; however, the boundaries between these segments were not clear, nor was there a distinct demarcation between the descending colon and the rectum. The bladder is located in the middle of the lower abdomen. Its shape is sac-like. Its ventral side is adjacent to the abdominal wall, and its dorsal side is adjacent to the rectum.

**Figure 3:**
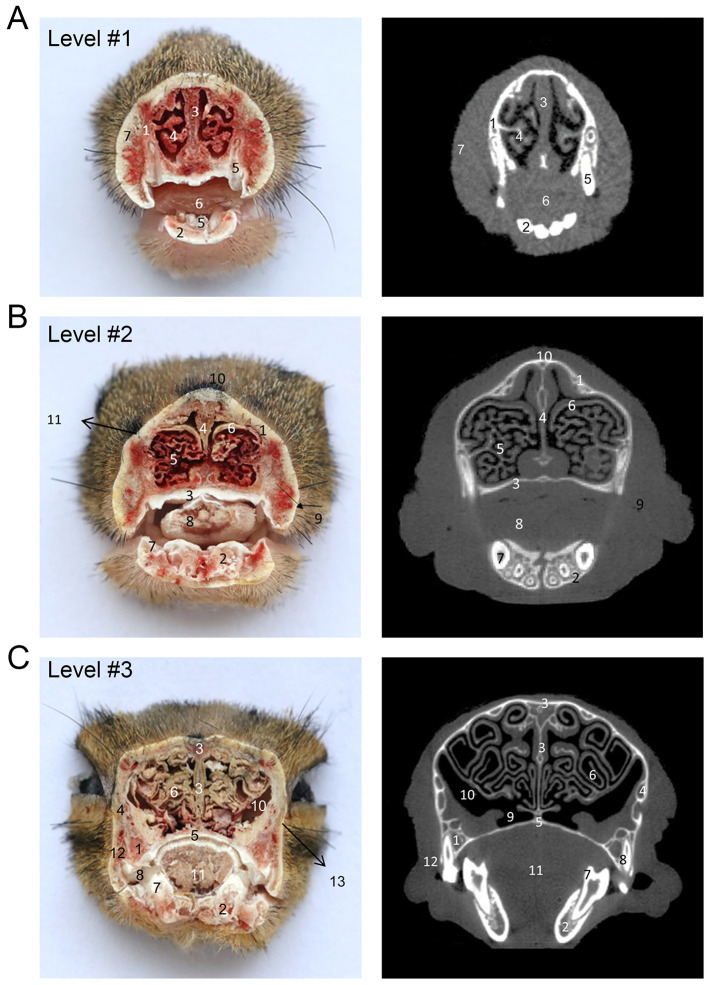
The cross-sectional anatomy and micro-CT imaging of the anterior head region. **(A)** 1. Maxilla; 2. Mandible; 3. Nasal septum; 4. Inferior nasal concha; 5. Incisors; 6. Tongue; 7. Scalp. **(B)** 1. Maxilla; 2. Mandible; 3. Palatine bone; 4. Nasal septum; 5. Inferior nasal concha; 6. First ethmoturbinate; 7. Canine teeth; 8. Tongue; 9. Nasal venous plexus; 10. Nasal bone; 11. Scalp. **(C)** 1. Maxilla; 2. Mandible; 3. Ethmoid bone; 4. Zygomatic bone; 5. Palatine bone; 6. Second ethmoturbinate; 7. Canine teeth; 8. Molar; 9. Common nasal meatus; 10. Maxillary sinus; 11. Tongue; 12. M. masseter; 13. Scalp.

**Figure 4:**
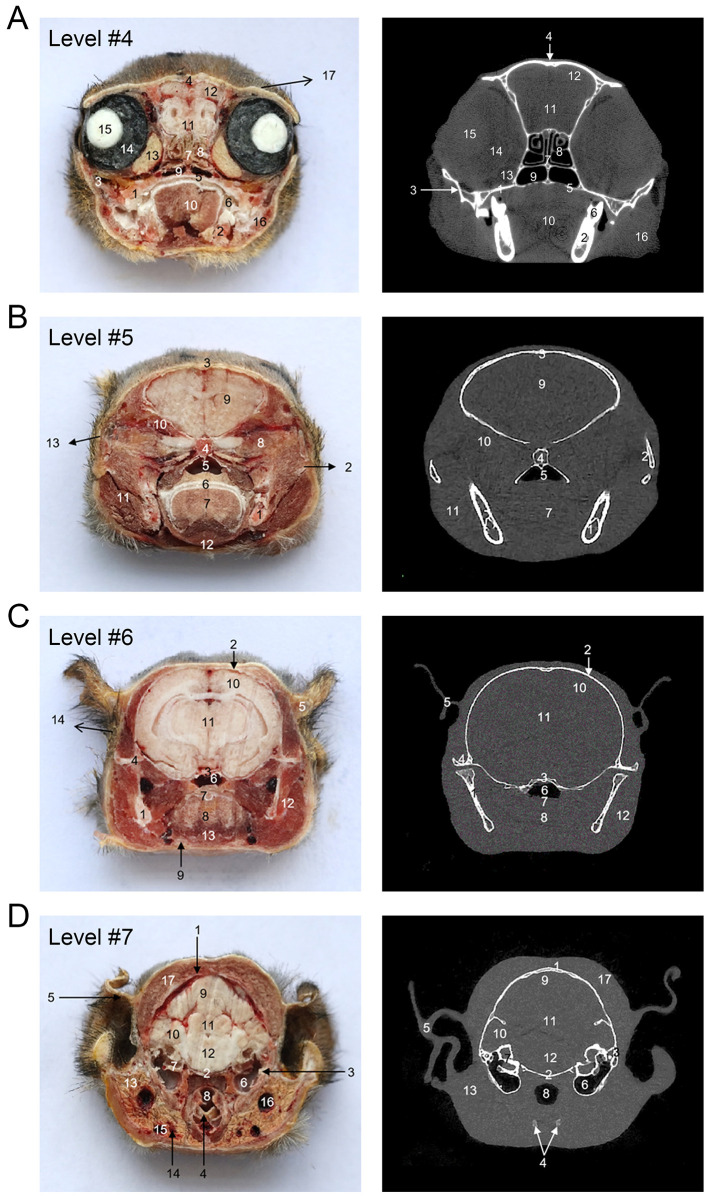
The cross-sectional anatomy and micro-CT imaging of the posterior head region. **(A)** 1. Maxilla; 2. Mandible; 3. Zygomatic bone; 4. Frontal bone; 5. Palate bone; 6. Molar; 7. Ethmoid bone; 8. Third ethmoturbinate; 9. Common nasal meatus; 10. Tongue; 11. Olfactory bulb; 12. Telencephalon; 13. Orbital adipose body; 14. Vitreous; 15. Lens; 16. M. masseter; 17. Scalp. **(B)** 1. Mandible; 2. Zygomatic bone; 3. Frontal bone; 4. Sphenoid bone; 5. Total nasal meatus; 6. Soft palate; 7. Tongue; 8. Orbital adipose body; 9. Cerebrum; 10. Temporalis muscle; 11. M. masseter; 12. M. digastricus; 13. Scalp. **(C)** 1. Mandible; 2. Parietal bone; 3. Sphenoid bone; 4. Zygomatic bone; 5. External ear; 6. Nasopharynx; 7. Soft palate; 8. Dorsum of tongue; 9. Sublingual gland; 10. Cerebrum; 11. Diencephalon; 12. M. masseter; 13. M. digastricus; 14. Scalp. **(D)** 1. Frontal bone; 2. Occipital bone; 3. Temporal bone; 4. Thyroid cartilage; 5. External ear; 6. Middle ear; 7. Inner ear; 8. Larynx; 9. Cerebrum; 10. Cerebellar cortex; 11. Cerebellum; 12. Pons; 13. Parotid gland; 14. A. facialis; 15. V. facialis; 16. V. retromandibularis; 17. M. occipitofrontalis.

**Figure 5:**
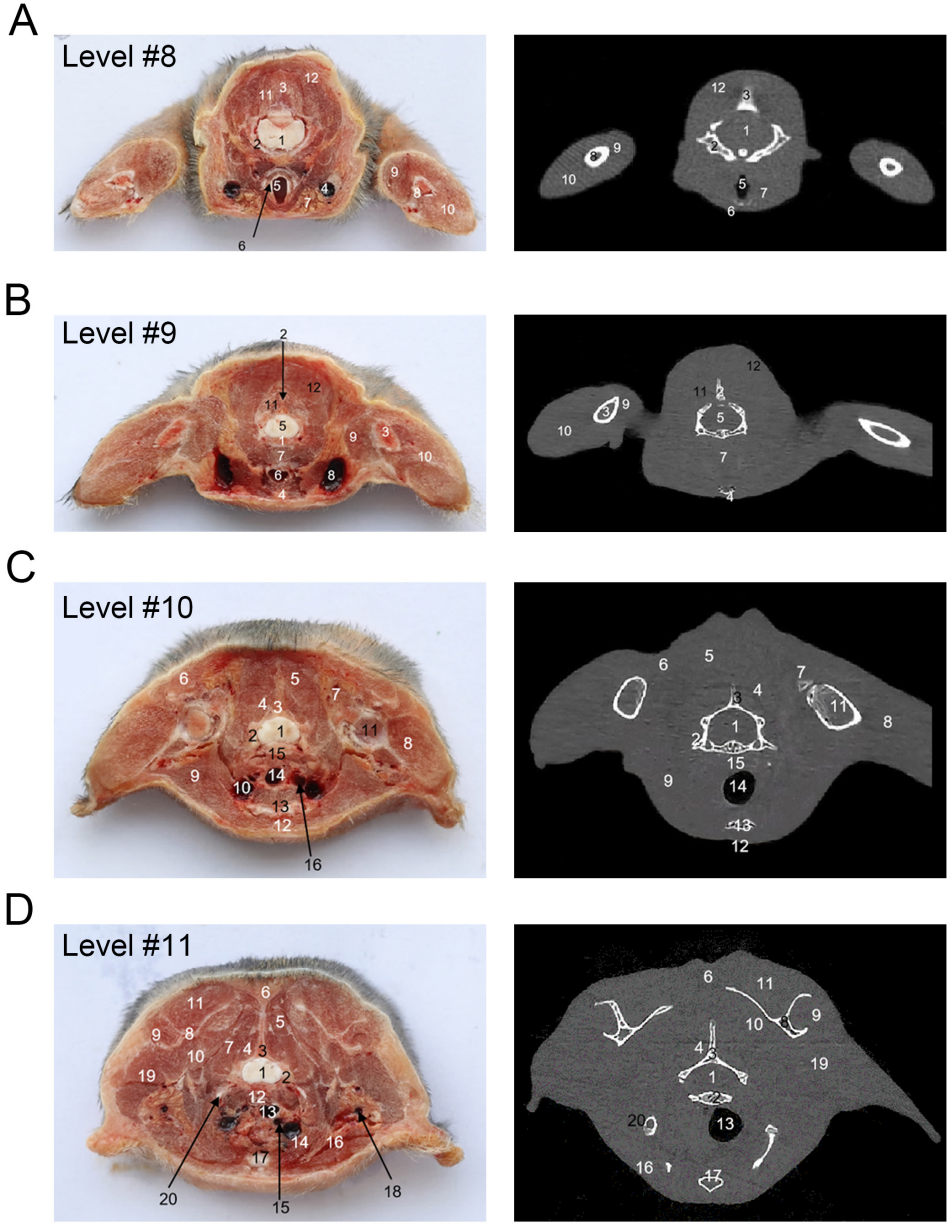
The cross-sectional anatomy and micro-CT imaging of neck and thorax region. **(A)** 1. Spinal cord; 2. Cervical vertebra (segment); 3. Cervical spinous process; 4. External jugular vein; 5. Trachea; 6. Thyroid cartilage; 7. Submandibular gland; 8. Humerus; 9. M. biceps brachii; 10. M. triceps brachii; 11. M. spinalis cervicis; 12. M. complexus. **(B)** 1. Cervical vertebrae; 2. Cervical spinous process; 3. Humerus; 4. Sternum; 5. Spinal cord; 6. Trachea; 7. Esophagus; 8. Subclavian vein; 9. M. biceps brachii; 10. M. triceps brachii; 11. M. spinalis cervicis; 12. M. complexus. **(C)** 1. Spinal cord; 2. Cervical vertebra; 3. Cervical spinous process; 4. M. spinalis cervicis; 5. M. complexus; 6. M. Trapezius; 7. M. biceps brachi; 8. M. triceps brachi; 9. M. subclavius; 10. V. subclavia; 11. Humerus; 12. M. rectus thoracis; 13. Sternum; 14. Trachea; 15. Esophagus; 16. A. carotis communis. **(D)** 1. Spinal cord; 2. Thoracic vertebra; 3. Thoracic spinous process; 4. Mm. multifidi; 5. M. spinalis thoracis; 6. M. trapezius; 7. M. serratus ventralis thoracis; 8. Scapula; 9. M. infraspinatus; 10. M. subscapularis; 11. M. supraspinatus; 12. Esophagus; 13. Trachea; 14. V. jugularis externa; 15. V. jugularis interna; 16. Mm. pectorales; 17. Sternum. 18. V. axillary; 19. M. teres major. 20. Rid.

**Figure 6:**
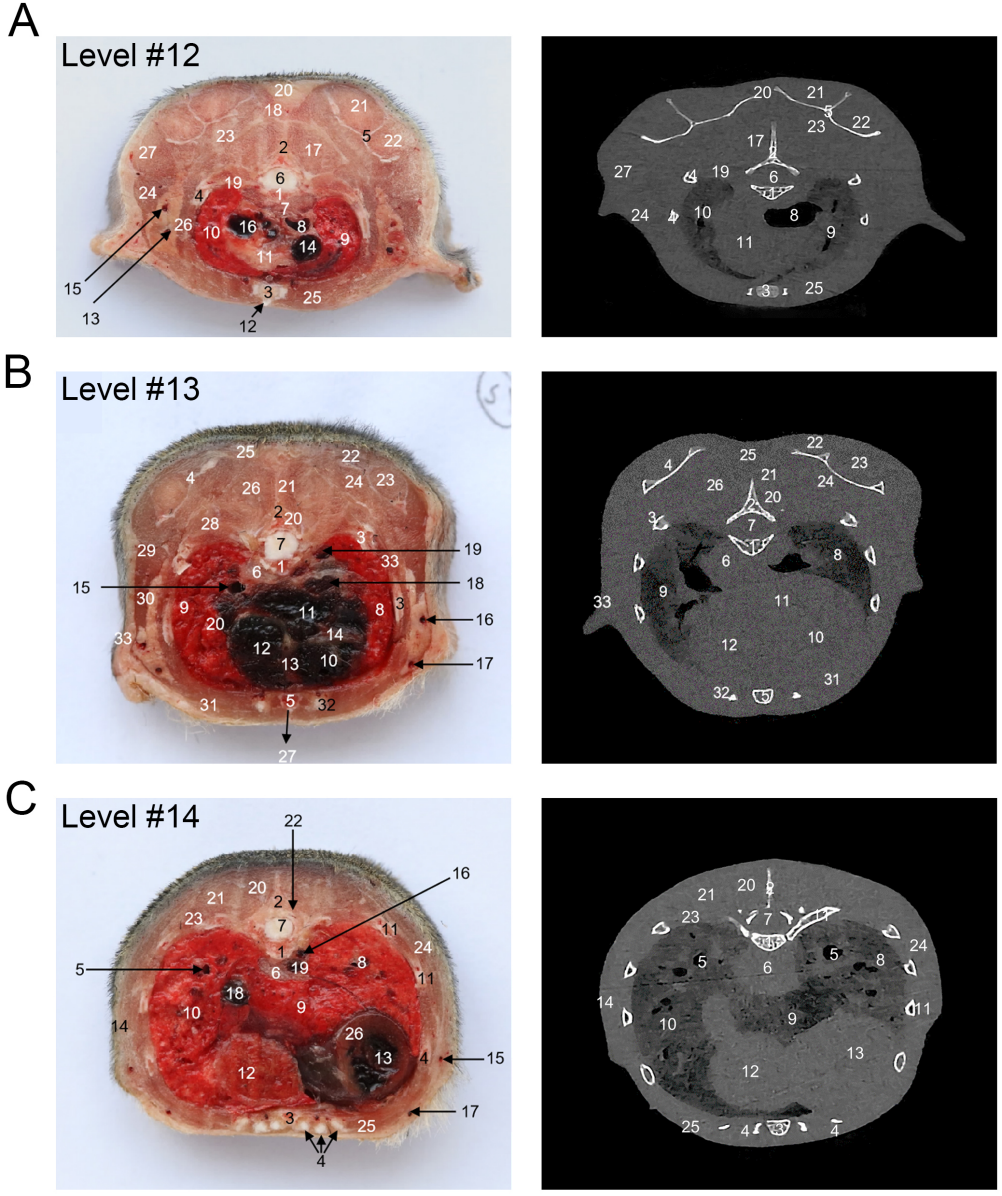
The cross-sectional anatomy and micro-CT imaging of thorax region. **(A)** 1. Thoracic vertebra; 2. Thoracic spinous process; 3. Sternum; 4. Rib; 5. Scapula; 6. Spinal cord; 7. Esophagus; 8. Trachea; 9. Pulmo sinister; 10. Right lung; 11. Thymus; 12. Linea alba; 13. V. thoracica superficialis; 14. V. cava anterior; 15. V. axillary; 16. A. pulmonalis dextra; 17. M. longissimus thoracis; 18. M. rhomboideus thoracis; 19. M. levator costae; 20. M. trapezius (pars thoracica); 21. M. infraspinatus; 22. M. supraspinatus; 23. M. subscapularis; 24. M. latissimus dorsi; 25. Mm. pectorales; 26. M. scalenus dorsalis; 27. M. teres major. **(B)** 1. Thoracic vertebra; 2. Thoracic spinous process; 3. Rib; 4. Scapula; 5. Sternum; 6. Esophagus; 7. Spinal cord; 8. Pulmo sinister; 9. Right lung; 10. Ventriculus dexter; 11. Ventriculus sinister; 12. Atrium dextrum; 13. Atrioventricular septum; 14. Interventricular Septum; 15. A. pulmonalis dextra; 16. V. axillary; 17. V. thoracica superficialis; 18. V. cava anterior; 19. A. thoracica; 20. Mm. multifidi; 21. M. spinalis thoracis; 22. M. supraspinatus; 23. M. infraspinatus; 24. M. subscapularis; 25. M. trapezius (parsthoracica); 26. M. longissimus thoracis; 27. Linea alba; 28. M. levator costae; 29. M. teres major; 30. M. latissimus dorsi; 31. Mm. pectorales; 32. Costal cartilage; 33. M. cutaneus trunci. **(C)** 1. Thoracic vertebra; 2. Thoracic spinous process; 3. Sternum; 4. Costal cartilage; 5. Lobar bronchi; 6. Esophagus; 7. Spinal cord; 8. Pulmo sinister; 9. Lung, accessory lobe; 10. Right lung; 11. Rib; 12. Middle hepatic lobe; 13. Ventriculus sinister; 14. M. cutaneus trunci; 15. V. axillary; 16. V. azygos; 17. V. thoracica superficialis; 18. V. cava caudalis; 19. A. thoracica; 20. M. spinalis thoracis; 21. M. longissimus thoracis; 22. Mm. multifidi; 23. M. levator costae; 24. M. latissimus dorsi; 25. Mm. pectorales; 26. Myocardium.

**Figure 7:**
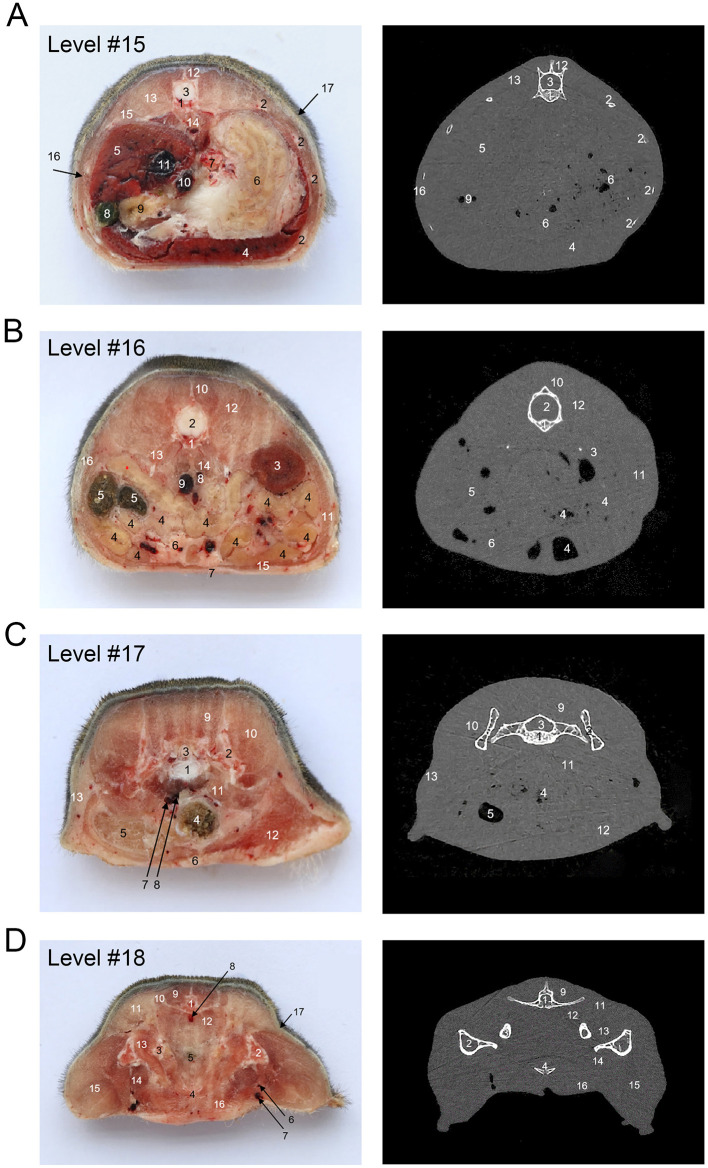
The cross-sectional anatomy and micro-CT imaging of abdomen region. **(A)** 1. Lumbar vertebrae; 2. Rib; 3. Spinal cord; 4. Liver, left lobe; 5. Liver, right lobe; 6. Stomach; 7. Ostium cardiacum; 8. Gallbladder; 9. Duodenum; 10. V. hepatica sin; 11. V. cava caudalis; 12. M. spinalis thoracis; 13. M. longissimus lumborum; 14. A. abdominalis; 15. M. iliocostalis thoracis; 16. M. latissimus dorsi; 17. M. cutaneus trunci. **(B)** 1. Lumbar vertebrae; 2. Spinal cord; 3. Ren sinister; 4. Jejunum; 5. Colon; 6. Mesenterium; 7. Linea alba; 8. A. thoracical; 9. V. cava caudalis; 10. Mm. multifidi; 11. M. obliquus externus abdominis + M. obliquus internus + M. transversus abdominis; 12. M. longissimus lumborum; 13. M. qudratus lumborum; 14. Mm. psoas; 15. M. rectus abdominis; 16. M. cutaneus trunci. **(C)** 1. Os sacrum; 2. Ilium; 3. Spinal cord; 4. Cecum; 5. Vesica urinaria. 6. Llinea alba; 7. V. cava caudalis; 8. A. thoracica; 9. Mm. multifidi; 10. M. longissimus lumborum; 11. Mm. psoas; 12. M. obliquus externus abdominis + M. obliquus internus + M. transversus abdominis; 13. M. cutaneus trunci. **(D)** 1. Os sacrum; 2. Femur; 3. Ilium; 4. Symphysis pubica; 5. Rectum; 6. Arteria femoralis; 7. Vena femoralis; 8. V. cava caudalis; 9. Mm. multifidi; 10. M. longissimus lumborum; 11. M. gluteus max; 12. Mm. psoas; 13. M. vastus medialis; 14. M. vastus intermedius; 15. M. vastus lateralis + M. rectus femoris; 16. M. rectus abdominis; 17. M. cutaneus trunci.

Figure 1 shows an X-ray horizontal view (scanogram) of the animal acquired immediately before the beginning of the CT-scan. The lines in Figure 1 indicate the approximate levels corresponding to each CT image and the anatomical slices of the cadaver. The results of our study are presented through matched photographs as shown in the figures. Figures 3–7 present cross-sectional anatomical images of the tree shrew from head to tail, along with the corresponding CT images. The identifiable anatomical structures are labeled on both the cadaver sections and the corresponding CT images. The asymmetry of the anatomical photographs resulted from slight deviations from the transverse plane during cutting. Figures 3, 4 show the cross-sectional anatomy of the head region, highlighting structures such as the skull, facial muscles, brain, and sensory organs. To ensure section uniformity, each slice was cut in a single pass. However, this generated considerable heat, especially when cutting bone, which caused thin parts (such as the ethmoid bone) to curl up (Figures 3B, C). Figure 5A presents the corresponding images of the neck, illustrating the neck muscles, blood vessels, and trachea. Figures 5B–D, 6 illustrate cross-sections of the thorax, displaying thoracic organs such as the heart, lungs, and major blood vessels. By examining sequential thoracic images, the identity of these blood vessels can be confirmed. Finally, Figure 7 depicts cross-sections of the abdomen, revealing digestive organs including the stomach, liver, intestines, and kidneys. The liver of tree shrew was large in size, rich in blood supply, brittle in texture after freezing, and occasionally cracked during cutting, and this was caused by man-made rather than vascular flow (Label 4 in Figure 7A). All cadaver photographs and their corresponding micro-CT images are presented in Figures 3–7.

## Discussion

In this study, we compared the micro-CT images with the cadaver cross-sectional photographs in light of the unique advantages offered by micro-CT. Each cross-section's anatomy could be clearly observed, and any morphological changes in the organs were accurately localized. CT provides good visualization of both hard and soft tissues, and it can produce static, highly detailed images of an entire specimen or a specific region. The cost of learning, using, and maintaining micro-CT is relatively low, which makes it easy for researchers to obtain imaging results. Consequently, this technology is now widely used in scientific research and clinical practice (13). Therefore, comparing CT images with anatomic slices can provide a more intuitive reference for researchers.

In this study, we used a slice thickness of 5 mm to maintain the integrity of the entire specimen. Micro-CT was employed to obtain higher-resolution scanned images. After the scanning was completed, the tree shrews were euthanized. Although we attempted to reposition the carcasses exactly as they were during scanning, it was difficult to achieve perfect alignment between the scanned images and the physical sections. Furthermore, freezing inevitably causes morphological changes in water-rich tissues. Imperfect alignment between cadaveric sections and micro-CT images constitutes an inherent methodological limitation that may especially affect the interpretation of thin or delicate structures. These differences stem primarily from variations in specimen positioning, discrepancies between section and slice thickness, and microstructural alterations introduced during freezing, embedding, and cutting processes. Although these factors may slightly influence the precision of spatial correspondence, the overall registration quality achieved in this dataset remains sufficient for the accurate identification of major anatomical features. Future technical developments, such as continuous submillimeter slicing or advanced 3D reconstruction technology, are expected to further improve alignment accuracy and mitigate misregistration artifacts (19, 20). Transparent recognition of this limitation enhances the scientific rigor of the present study and clarifies its scope of applicability. Despite these constraints, the dataset retains high value as a fundamental anatomical reference for integrative imaging research.

Due to the technical complexity of micro-CT scanning and anatomical sectioning, only two specimens were used for detailed image correlation to ensure the highest image quality and registration between the micro-CT data and the corresponding gross anatomical sections. However, anatomical consistency was verified across all four specimens to confirm the reproducibility of observed structures. Because it may not encompass the full range of normal anatomical variation within the species, the small sample size underlying the present atlas represents a methodological limitation. This atlas provides a valuable high-resolution morphological benchmark for tree shrew anatomy and is intended to serve as a foundational reference resource. Future research should build upon this atlas by including larger cohorts in order to systematically quantify inter-individual anatomical variability.

The integration of micro-CT imaging with cadaveric sectioning provided substantial methodological advantages for the anatomical characterization of the tree shrew, a small mammalian species with intricate soft-tissue architecture. Owing to the narrow spatial intervals and fine structural differentiation in this species, micro-CT alone is limited in resolving thin membranous or overlapping structures, whereas isolated cadaveric sections may lack the three-dimensional continuity necessary for spatial localization. By combining both modalities, the present study achieved improved anatomical resolution and interpretative accuracy. The integrated micro-CT–cadaver framework thus provides a robust, species-specific anatomical reference for the tree shrew, enabling more accurate correlation between imaging features and morphological reality, and establishing a methodological foundation for translational imaging research in this model organism. The anatomical atlas provided by this study will help researchers identify tree shrew anatomical structures in specific regions of interest, and it can also serve as a valuable reference for future anatomical studies involving tree shrews.

Current anatomical studies of the tree shrew predominantly focus on specific regional structures, such as the temporal bone, nasal anatomy, and neuroanatomy (17, 21, 22). These include MRI-based brain atlases that provide high-resolution intracranial templates, as well as detailed cerebellar atlases offering systematic nomenclature, histochemical characterization, and connectivity mapping of cerebellar lobules (23, 24). While these studies provide precise anatomical definitions, they lack a comprehensive whole-body spatial context. In contrast, the present study offers continuous whole-body coverage in standardized transverse planes, enabling simultaneous visualization of skeletal landmarks and visceral organs, thereby supplementing existing anatomical research. Future investigations could benefit from integrating histopathology, microstructural imaging, and other advanced modalities into a multi-scale anatomical platform. Such integration would expand the experimental utility of the tree shrew and strengthen its translational relevance in modeling a wide spectrum of human diseases.

## Data Availability

The original contributions presented in the study are included in the article/supplementary material, further inquiries can be directed to the corresponding authors.

## References

[1] Cao J Yang EB Su JJ Li Y Chow P. The tree shrews: adjuncts and alternatives to primates as models for biomedical research. J Med Primatol. (2003) 32:123–30. doi: 10.1034/j.1600-0684.2003.00022.x 12823622

[2] Fan Y Yu D Yao YG. Genome of the Chinese tree shrew. Nat Commun. (2013) 4:1426. doi: 10.1038/ncomms2416 23385571

[3] Shen PQ Zheng H Liu RW Chen LL Li B He BL . Progress and prospect in research on laboratory tree shrew in China. Dongwuxue Yanjiu. (2011) 32:109–14. doi: 10.3724/SP.J.1141.2011.01109 21341393

[4] Xu L Zhang Y Liang B Lü LB Chen CS Chen YB . Tree shrews under the spot light: emerging model of human diseases. Dongwuxue Yanjiu. (2013) 34:59–69. doi: 10.3724/SP.J.1141.2013.02059 23572354

[5] Bowen DG. Toward small animal models for the study of human hepatitis viruses. Hepatology. (2010) 52:382–4. doi: 10.1002/hep.23755 20583194

[6] Li R Yuan B Xia X Zhang S Du Q Yang C . Tree shrew as a new animal model to study the pathogenesis of avian influenza (H9N2) virus infection. Emerg Microbes Infect. (2018) 7:166. doi: 10.1038/s41426-018-0167-1 30301950 PMC6177411

[7] Qi K Feng M Meng X Li Y Zhu N Sui N. Social defeat paradigm in tree shrews as a depression model. Adv Psychol Sci. (2012) 20:1787–93. doi: 10.3724/SP.J.1042.2012.01787

[8] Ruan GP Yao X Liu JF He J Li ZA Yang JY . Establishing a tree shrew model of systemic lupus erythematosus and cell transplantation treatment. Stem Cell Res Ther. (2016) 7:121. doi: 10.1186/s13287-016-0385-1 27558022 PMC4995612

[9] Wang J Zhou QX Lv LB Xu L Yang YX. A depression model of social defeat etiology using tree shrews. Dongwuxue Yanjiu. (2012) 33:92–8. doi: 10.3724/SP.J.1141.2012.01092 22345016

[10] Ye L He M Huang Y Zhao G Lei Y Zhou Y . Tree shrew as a new animal model for the study of lung cancer. Oncol Lett. (2016) 11:2091–5. doi: 10.3892/ol.2016.4156 26998127 PMC4774532

[11] Bai X Yu L Liu Q Zhang J Li A Han D . A high-resolution anatomical rat atlas. J Anat. (2006) 209:707–8. doi: 10.1111/j.1469-7580.2006.00645.x 17062027 PMC2100338

[12] Karlon WJ Eisenberg SR Lehr JL. Effects of paddle placement and size on defibrillation current distribution: a three-dimensional finite element model. IEEE Trans Biomed Eng. (1993) 40:246–55. doi: 10.1109/10.216408 8335328

[13] Samii VF Biller DS Koblik PD. Normal cross-sectional anatomy of the feline thorax and abdomen: comparison of computed tomography and cadaver anatomy. Vet Radiol Ultrasound. (1998) 39:504–11. doi: 10.1111/j.1740-8261.1998.tb01640.x 9845186

[14] Zotti A Banzato T Cozzi B. Cross-sectional anatomy of the rabbit neck and trunk: comparison of computed tomography and cadaver anatomy. Res Vet Sci. (2009) 87:171–6. doi: 10.1016/j.rvsc.2009.02.003 19298990

[15] Dai JK Wang SX Shan D Niu HC Lei H. Super-resolution track-density imaging reveals fine anatomical features in tree shrew primary visual cortex and hippocampus. Neurosci Bull. (2018) 34:438–48. doi: 10.1007/s12264-017-0199-x 29247318 PMC5960443

[16] Li B Zhang RP Li JT He BL Zhen H Wang LM . Measurement and analysis of anatomical parameter values in tree shrews. Dongwuxue Yanjiu. (2013) 34:132–8. doi: 10.3724/SP.J.1141.2013.02132 23572364

[17] Lihong X Heng L Gyanwali B Meichan Z Kaiquan Z Kai S . Micro-computed tomography and microdissection of the temporal bone of tree shrews. Ann Anat. (2016) 208:69–77. doi: 10.1016/j.aanat.2015.08.005 26409819

[18] Tanzola R. Sonoanatomy for anaesthetists. Can J Anaesth. (2013) 60:830–1. doi: 10.1007/s12630-013-9970-2

[19] Abreu de Souza M Alka Cordeiro DC Oliveira J Oliveira MFA Bonafini BL. 3D Multi-modality medical imaging: combining anatomical and infrared thermal images for 3D reconstruction. Sensors. (2023) 23:1610. doi: 10.3390/s23031610 36772650 PMC9919921

[20] Chang S Li L Hong B Liu J Xu Y Pang K . An intelligent workflow for sub-nanoscale 3D reconstruction of intact synapses from serial section electron tomography. BMC Biol. (2023) 21:198. doi: 10.1186/s12915-023-01696-x 37743470 PMC10519085

[21] Feng Y Xia W Zhao P Yi X Tang A. Survey anatomy and histological observation of the nasal cavity of *Tupaia belangeri* Chinensis (Tupaiidae, Scandentia, Mammalia). Anat Rec. (2022) 305:1448–58. doi: 10.1002/ar.24793 34605617

[22] Huang Q Nie B Ma C Wang J Zhang T Duan S . Stereotaxic (18)F-FDG PET and MRI templates with three-dimensional digital atlas for statistical parametric mapping analysis of tree shrew brain. J Neurosci Methods. (2018) 293:105–16. doi: 10.1016/j.jneumeth.2017.09.006 28917660

[23] Ni RJ Huang ZH Luo PH Ma XH Li T Zhou JN. The tree shrew cerebellum atlas: systematic nomenclature, neurochemical characterization, and afferent projections. J Comp Neurol. (2018) 526:2744–75. doi: 10.1002/cne.24526 30155886

[24] Wang S Shan D Dai J Niu H Ma Y Lin F . Anatomical MRI templates of tree shrew brain for volumetric analysis and voxel-based morphometry. J Neurosci Methods. (2013) 220:9–17. doi: 10.1016/j.jneumeth.2013.08.023 24012828

